# Selected Saliva-Derived Cytokines and Growth Factors Are Elevated in Pediatric Dentofacial Inflammation

**DOI:** 10.3390/ijms25168680

**Published:** 2024-08-09

**Authors:** Bogusława Orzechowska-Wylęgała, Adam Wylęgała, Jolanta Zalejska Fiolka, Zenon Czuba, Katarzyna Kryszan, Michał Toborek

**Affiliations:** 1 Department of Pediatric Otolaryngology, Head and Neck Surgery, Medical University of Silesia (SUM), 40-055 Katowice, Poland; 2 Health Promotion and Obesity Management, Department of Pathophysiology, 40-055 Katowice, Poland; 3 Department of Biochemistry, Faculty of Medical Science in Zabrze, Medical University of Silesia, 40-055 Katowice, Poland; 4 Department and Division of Microbiology and Immunology in Zabrze, Medical University of Silesia, 41-800 Zabrze, Poland; 5 Chair and Clinical Department of Ophthalmology, School of Medicine in Zabrze, Medical University of Silesia in Katowice, District Railway Hospital, 40-760 Katowice, Poland; 6 Department of Biochemistry and Molecular Biology, University of Miami School of Medicine, Miami, FL 33136, USA

**Keywords:** cytokines, growth factors, saliva, dentofacial structures, inflammation, dental caries, child

## Abstract

Dentofacial inflammation resulting from untreated dental caries is a serious disease that can spread to deeper tissues of the neck and face. This study aimed to analyze salivary cytokine profiles as potential biomarkers of acute odontogenic infections in children. The study group consisted of 28 children aged 3–17 years old with acute dentofacial infections (DI) and a control group (caries experience, CE) of 52 children aged 4–17 years old with uncomplicated dental caries. The cytokine profile was analyzed using the Bio-Plex Pro Human Cytokine 27-Plex kit in the saliva of children in both groups. The levels of IL-4, IL-15, FGF-2, G-CSF, and PDGF-BB were significantly increased in children with dentofacial infections compared to the control group. In contrast, the levels of other cytokines, such as IL-2, IL-7, IL-9, IL-13, GM-CSF, and IFN-γ, did not show statistically significant differences between these two groups. IL-4, IL-15, FGF-2, G-CSF, and PDGF-BB may serve as potential selective biomarkers of inflammation of the oral cavity in children. These biomarkers can be useful in identifying and monitoring the progress and treatment of bacterial infections resulting in dentofacial inflammation.

## 1. Introduction

Dentofacial inflammation in children and adolescents is a significant clinical issue due to its rapid spread through blood vessels and lymphatics in the head and neck region. This inflammation, originating from dental infections, necessitates immediate treatment to prevent further complications [1]. Factors such as low jawbone calcification, large marrow spaces, developing teeth, an underdeveloped maxillary sinus, and a higher proportion of spongy bone contribute to the rapid progression of inflammation. Children’s unique immune responses also play a role, leading to potential complications like swelling, pain, and difficulty swallowing or breathing [2].

Unfavorable conditions such as metabolic irregularities, mucous membrane injuries, or immune disturbances can disrupt the balance of oral bacterial flora, turning commensal bacteria into opportunistic pathogens. This can lead to severe infections, including submucosal abscesses, life-threatening space infections, bone and marrow inflammation, meningitis, or maxillary sinus thrombophlebitis [3].

The treatment approach for odontogenic inflammatory connective tissue in children involves tooth removal or endodontic treatment, abscess drainage if necessary, and antibiotic therapy [4]. It is crucial to consider anatomical, pathophysiological, and pharmacokinetic differences between pediatric and adult patients during treatment [5]. While odontogenic cellulitis in adults is often polymicrobial with significant anaerobic bacterial components, in children, staphylococci and streptococci are the most common pathogens [6,7].

Recent advancements focus on the natural defense system of the oral cavity, particularly saliva’s role in maintaining oral health [2]. Saliva is essential for preserving the structural integrity of both hard and soft tissues within the oral cavity. Unlike blood collection, saliva collection is non-invasive and beneficial for children, providing comprehensive analyses of cytokines and chemokines crucial for stimulating the immune system to combat infection and inflammation [8,9].

This study focuses on the analysis of specific saliva cytokines, including interleukins (ILs) responsible for cellular mechanisms such as interleukin-2 (IL-2), interleukin-4 (IL-4), interleukin-7 (IL-7), interleukin-9 (IL-9), interleukin-13 (IL-13) interleukin-15 (IL-15), and growth factors such as fibroblast growth factor 2 (FGF-2, FGF-basic), granulocyte colony-stimulating factor (G-CSF), granulocyte–macrophage colony-stimulating factor (GM-CSF), platelet-derived growth factor-BB (PDGF-BB), and interferon-gamma (IFN-γ) [10,11]. These interleukins and growth factors are all involved in regulating various aspects of immune function, tissue repair, and inflammation, highlighting their importance in maintaining overall health and homeostasis (Table 1) [3,12].

There is an emerging need to conduct further investigation into the influence of saliva-based cytokines on the inflammatory reactions associated with dentofacial infections in children. By clarifying the intricate molecular pathways involved in these responses, future research endeavors promise to identify innovative diagnostic tools and therapeutic interventions to address these infections better and enhance oral health outcomes in pediatric patients.

By aiming to characterize a profile of salivary cytokine changes in children, our study addresses a significant void in the existing literature by exploring the significance of salivary biomarkers in pediatric dentofacial infections. We aspire to provide novel perspectives on developing and treating these conditions, thereby advancing both the scientific knowledge base and the standards of clinical care in pediatric dentistry. The results suggest that a selective group of salivatory cytokines may serve as potential biomarkers for children’s acute odontogenic oral and facial infections.

## 2. Results

In our cohort, we identified a subset of 28 children diagnosed with acute dentofacial infections (DIs), comprising 7 girls and 21 boys aged 3 to 17 years, with a mean age of 8.67 years (SD ± 4.64). The control group (caries experience, CE) consisted of 52 children, including 16 girls and 36 boys aged 4 to 17 years, with a mean age of 8.38 years (SD ± 3.67), who presented with uncomplicated dental cavities.

In 17 cases, the causes of dentofacial inflammation of the connective tissue in the face and neck were primary teeth, while in 11 cases, permanent teeth were the cause. Among primary teeth, the majority of inflammatory processes originated from the upper teeth in 13 cases, compared to permanent dentition, where lower teeth predominated in 7 cases. Detailed data are presented in Table 2.

**Figure 1:**
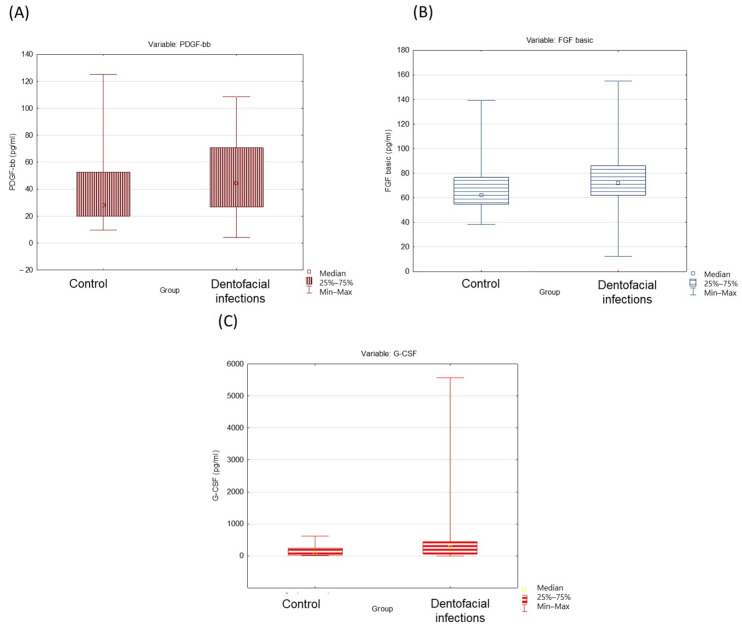
Box-and-whisker plots depicting platelet-derived growth factor-BB (PDGF-bb) (**A**), fibroblast growth factor (FGF-basic) (**B**), and granulocyte colony-stimulating factor (G-CSF) (**C**).

Our analyses revealed statistically significant changes between the studied groups among several growth factors studied. The median cytokine PDGF-BB level (Figure 1A) for individuals with CE was 27.91, while for those with DI, the value significantly increased to 44.28 (*p =* 0.04). Similarly, FGF-basic levels were elevated in individuals with DI compared to those in the CE group (62.15 vs. 71.94, *p =* 0.04) (Figure 1B). Additionally, G-CSF levels exhibited a significant elevation in individuals with DI compared to those with CE (81.47 vs. 269.28, *p =* 0.02) (Figure 1C).

**Figure 2:**
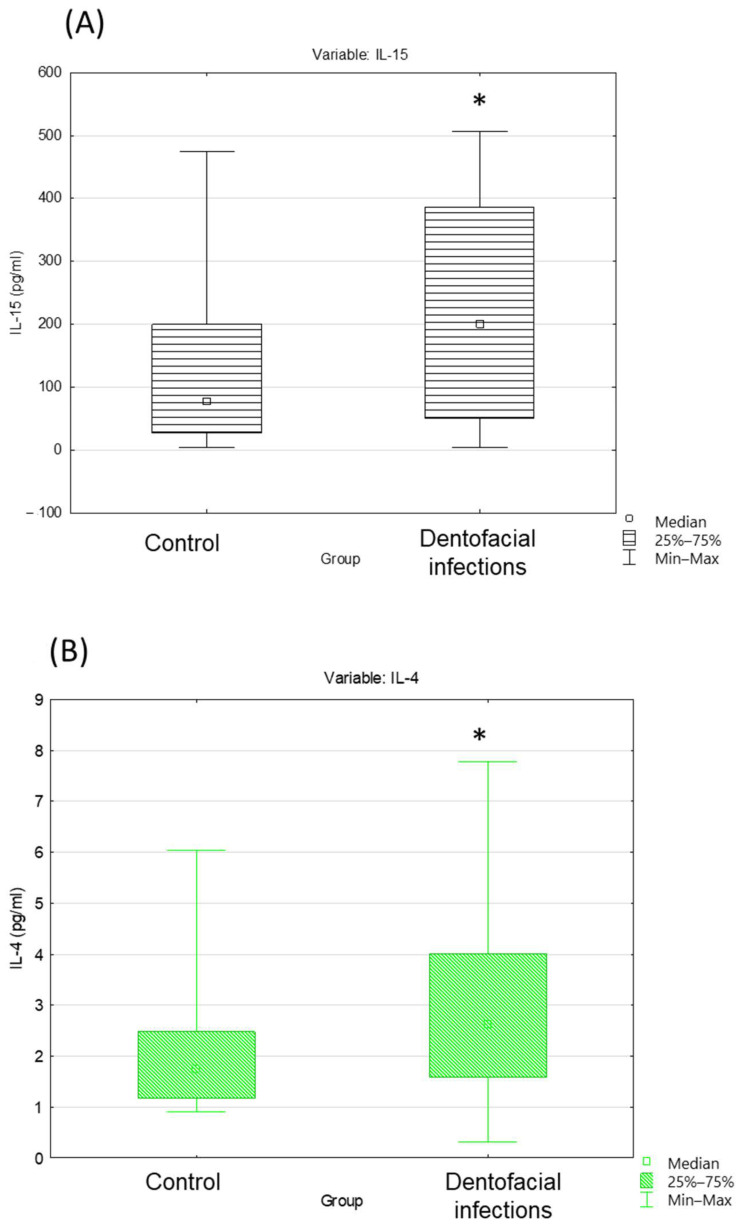
Box-and-whisker plots depicting interleukin-15 (IL-15) (**A**) and interleukin-4 (IL-4) (**B**). The box represents the 25–75% range, the square inside the box represents the median, and the whiskers represent the minimum to maximum range. * *p* < 0.05.

Regarding the cytokine levels, IL-15 levels demonstrated a considerable elevation in individuals with DI compared to those with CE (77.96 vs. 199.85, *p =* 0.04) (Figure 2A). Similarly, IL-4 levels showed a notable increase in individuals with DI compared to those with CE (1.74 vs. 2.63, *p =* 0.03) (Figure 2B).

In contrast, the levels of IL-2, IL-7, IL-9, IL-13, GM-CSF, and IFN-γ did not show statistically significant differences between the two groups (Table 3).

## 3. Discussion

The objective of this study was to assess the concentrations of interleukins and growth factors in the saliva of pediatric patients affected by dentofacial infections. This issue holds significant clinical relevance, given that inflammation in the oral and facial regions can arise from various prevalent dental ailments, including tooth infections, abscesses, and periodontal diseases. Notably, there is a scarcity of literature addressing this specific area, highlighting the pioneering nature of our research endeavor in this field. The lack of available literature may stem from the straightforward detection of dental caries through clinical and radiographic assessments, as only Rinderknecht et al. assessed salivary cytokines and oral health in the pediatric population [19]. However, it is crucial to identify specific biomarkers of dentofacial infections as essential prognostic and preventive indicators for both caries and the potential complications associated with this condition. These proteins play crucial roles in the immune response and the regulation of inflammation within the body, being involved in processes such as chemotaxis, immune cell recruitment, and angiogenesis. Numerous reviews have detailed the evidence supporting salivary cytokines as biomarkers for oral conditions. Studying these biomarkers can enhance our understanding of pediatric dentofacial inflammation and inform effective preventive and therapeutic approaches [3,12,20,21].

In the premise of our publication, the control group consisted of children with moderate caries, whom we treated as the group before the onset of dentofacial infection. Children in the control group, except INF-γ, had lower levels of all the parameters tested, which demonstrates that acute inflammation in the head and neck region significantly influences the increase in the measured proteins in saliva. The findings of our investigation suggest that saliva serves as a valuable medium for exploring biomarkers that could influence the onset of acute dentofacial infections in pediatric patients.

IL-2, IL-4, IL-7, IL-9, IL-15, and IFN-γ are cytokines typically associated with proinflammatory responses, but IL-13 is an anti-inflammatory cytokine. Growth factors, such as FGF-basic, G-CSF, GM-CSF, and PDGF-BB, play crucial roles in stimulating immune responses, promoting inflammation, and regulating various cellular processes involved in immune defense and tissue repair [16,17].

Among the six interleukins measured, only IL-4 and IL-15 showed a significantly higher level in the group of children with dentofacial tissue inflammation compared to the group with uncomplicated dental caries. Th2 lymphocytes play a role in the production of IL-4 and IL-13, which in turn contribute to the formation of M2 macrophages known for their anti-inflammatory properties. The increase in salivary Th2 cytokine (IL-4) may indicate an adaptive response of the salivary glands to local inflammation in children [20,22,23].

Out of the four growth factors assessed, three exhibited statistically significant increases in these groups. FGF-basic is a member of the fibroblast growth factor family. It plays a crucial role in angiogenesis, wound healing, and tissue regeneration. FGF-basic stimulates the proliferation and differentiation of various cell types, including endothelial cells, fibroblasts, and neural progenitor cells.

G-CSF is a cytokine that regulates the production, differentiation, and function of neutrophils, a type of white blood cell involved in the immune response against bacterial and fungal infections. G-CSF is commonly used clinically to stimulate the production of neutrophils in patients undergoing chemotherapy or bone marrow transplantation [24]. 

PDGF-BB is a member of the platelet-derived growth factor family, which plays a central role in wound healing, tissue repair, and the regulation of cell proliferation and migration. PDGF-BB is primarily produced by platelets, endothelial cells, and macrophages and acts on various cell types, including fibroblasts, smooth muscle cells, and endothelial cells [25,26].

The results of this study indicate that detecting IL-4, IL-15, FGF-basic, G-CSF, and PDGF-BB can serve as reliable biomarkers for assessing inflammatory conditions within the oral cavity and facial regions among children. Similar results were described by Szulimowska et al., who evaluated the full 27 inflammatory and anti-inflammatory profiles in the saliva of children with chronic kidney disease (CKD) [12]. In our other article, we assessed cytokines IL-5, IL-10, IL-17A, IL-12p70, chemokines Eotaxin and Rantes, and growth factor VEGF. Among them, only IL-10 and IL-17A showed a significantly higher level in the group of children with dentofacial tissue inflammation compared to the group with uncomplicated dental caries [3,12].

It is important to acknowledge the limitations of this study. One significant limitation was the relatively small sample size of children with dentofacial infections, even though it met the requirements for statistical analysis as determined by sample size calculations. The hospital where we work is exclusively pediatric. We treated only children up to the age of 18, which is why adult patients were excluded from this study. Furthermore, we did not consider the effect of diet or oral hygiene practices on the measured parameters. Secondly, this study did not include a comparison group of healthy children without any dental issues. Additionally, only children without underlying general health issues were included in this study, allowing for a more accurate evaluation of salivary gland function in dentofacial infections. As the assessment of inflammatory and anti-inflammatory factors in unstimulated saliva primarily reflects the secretory activity of the submandibular glands, there is a need for additional research to explore the inflammatory profile in stimulated saliva, as well as in blood (plasma/serum) and through in vitro and in vivo models. While these areas hold promise, extensive research is necessary to validate these findings and translate them into safe and effective clinical treatments. These approaches offer a glimpse into the future of medicine; extensive clinical trials and safety assessments are crucial before widespread adoption.

## 4. Materials and Methods

### 4.1. Study Groups

This study was performed between 2020 and 2022 in the Division of Pediatric Laryngology, Head and Neck Surgery of the Department of Pediatric Surgery at the Silesian Medical University in Katowice, Poland. The primary objective was to explore the prevalence and potential indicators of acute inflammation in the oral and facial regions among children. This study encompassed two distinct groups: one with uncomplicated caries and another group with dentofacial infections.

Diagnoses of inflammatory conditions related to dental issues adhered to the criteria outlined by the World Health Organization (WHO), encompassing clinical, radiographic, and laboratory factors for identifying and categorizing oral and dental ailments. Adherence to WHO criteria provided a standardized methodology for diagnosis, which could facilitate treatment planning and disease monitoring over time. Ethical approval for this study was granted by the Bioethical Committee of the Medical University of Silesia under reference number PCN/0022/KB1/1/20.

### 4.2. Inclusion Criteria 

Entailed children with acute dentofacial infections devoid of systemic ailments and recent medication intake. 

### 4.3. Exclusion Criteria 

Included systemic conditions impeding study continuation, lack of child cooperation, and parental refusal _to_ participate. 

All legal guardians and children above 16 years old provided informed consent for participation.

Children with dentofacial infections presented with their parents to the Hospital Emergency Department, where they were admitted to the Pediatric Otolaryngology Ward. The following day, they remained fasting to undergo oral cavity sanitation procedures and potential drainage of abscess or cellulitis. Saliva samples were collected from them before the testing procedure. 

Children in the control group with uncomplicated caries presented with their parents to the Maxillofacial Surgery Outpatient, where they were referred to the Short-Stay Surgery Unit. On the scheduled day, they arrived fasted for saliva collection and underwent oral cavity sanitation procedures.

Examinations were conducted by a single practitioner, B.O.W., initially through visual and tactile assessments, followed by intra-oral examinations utilizing probes and mirrors. For the dentofacial infection group, the number of carious teeth and those causing inflammation were determined, while in the control group, the count focused on teeth with uncomplicated cavities.

Saliva samples were collected in the morning between 8:00 and 11:00 AM on an empty stomach post-mouth rinsing with water and a 10 min wait period. Patients were instructed to self-collect 1.5 mL of their unstimulated non-diluted saliva into a sterile tube, ensuring they avoided mucous secretions from the oropharynx and sputum. Samples were subsequently centrifuged for 10 min at 3000 rpm at 4 °C using a Centurion centrifuge and stored at −80 °C for future analysis. A rack containing 96 coded test tubes, each 1.2 mL and made of PP material (BRAND cat. no. 781566), was used for saliva collection. The analysis of these samples was conducted within six months from the time of freezing.

The significance of this procedure was communicated to parents and older children involved in this study.

### 4.4. Assessment of Immune Mediators

The evaluation of immune mediators involved the measurement of cytokine levels utilizing the Bio-Plex 200 System by Bio-Rad and the Bio-Plex Pro Human Cytokine Grp I Panel 27-Plex kit by the manufacturer’s guidelines (Warsaw, Poland). These assessments were conducted at the Department of Microbiology and Immunology in Zabrze, within the Medical University of Silesia in Katowice. All procedures adhered to good laboratory practice (GLP) standards. To ensure impartiality, samples were anonymized and assigned numerical identifiers. Additionally, all analytical techniques underwent continual interlaboratory quality control checks and met the standards set by external (interlaboratory) controls coordinated by the Central Center for Quality Testing in Laboratory Diagnostics in Łódź, Poland, and Labquality in Finland [18,24,27].

### 4.5. Statistical Analysis

The statistical analysis was conducted using Statistica 13 software (Tibco, Palo Alto, CA, USA). The Shapiro–Wilk test was used to check for normality. Student’s *t*-test was employed for parametric samples (IL-7), while the Mann–Whitney *U* test was utilized for nonparametric samples (all other cytokines).

The determination of the study’s sample size was derived from the mean and standard deviation documented in the research paper authored by Menon et al. [13]. The significance level was set at *p* ≤ 0.05, with a desired power of 90%. Based on these parameters, a sample size of 24 patients per group was calculated, ensuring a statistical power of 90.9% to attain significant findings.

## 5. Conclusions

This research proposes that evaluating specific proinflammatory cytokines like IL-4, IL-15, FGF-basic, G-CSF, and PDGF-BB can be reliable biomarkers for assessing inflammatory conditions within the oral cavity and facial regions among children.

## Figures and Tables

**Table 1:** Cytokine and growth factor production and primary functions [13,14,15,16,17,18].

Cytokines	Produced by	Primary Functions
IL-2: Interleukin-2	Activated T cells	Proliferation and differentiation of T cells, B cells, NK cells, and other immune cells
IL-4: Interleukin-4	T cells, mast cells, and basophils	Promotes Th2 cell differentiation, stimulates B cell proliferation, and enhances immunoglobulin class switching to IgE
IL-7: Interleukin-7	Stromal cells in bone marrow and thymus	Development and homeostasis of T cells, and promoting survival, proliferation, and differentiation of T cells
IL-9: Interleukin-9	Th9 cells and other immune cells (to a lesser extent)	Promotes survival and proliferation of mast cells and enhances function of Th2 cells
IL-13: Interleukin-13	Th2 cells, mast cells, and basophils	Promotes allergic responses, is involved in tissue repair, and regulates immune cell function
IL-15: Interleukin-15	Monocytes, macrophages, and dendritic cells	Development, survival, and activation of NK cells and memory CD8+ T cells and promoting proliferation of T cells
GM-CSF: Granulocyte–Macrophage Colony-Stimulating Factor	Various immune cells	Stimulates production and function of granulocytes (neutrophils, eosinophils, basophils) and macrophages, plays a role in immune response against infections, and regulates inflammatory processes
IFN-γ: Interferon-Gamma	Activated T cells and NK cells	Enhances antimicrobial activity of macrophages, promotes differentiation of Th1 cells, and modulates adaptive immune responses
PDGF-BB: Platelet-Derived Growth Factor-BB	Thrombocytes	Cell proliferation, wound healing, development of various organs and tissues during embryogenesis, and regulation of blood flow
FGF-basic: Basic Fibroblast Growth Factor	Fibroblasts, epithelial cells, endothelial cells, and tumor cells	Stimulator of angiogenesis, wound healing, repair of various tissues, and embryonic development
G-CSF: Granulocyte Colony-Stimulating Factor	Macrophages, fibroblasts, T lymphocytes, and endothelial cells	Stimulates white blood cell production, neutropenia treatment, and stem cell transplantation

**Table 2:** Causes of cellulitis in the dentofacial infection group (DI).

Primary Teeth				
	maxilla		mandible	
	right	left	right	left
Number	7	6	2	2
Total	13		4	
Permanent teeth				
	maxilla		mandible	
	right	left	right	left
number	1	3	2	5
Total	4		7	

**Table 3:** Comparative Analysis of Cytokine Expression Levels about Caries Experience and Dental Infections.

Variable	Caries Experience (CE)					Dental Infections (DIs)			
	Median	Minimum	Maximum	Standard	*p*-Value	Median	Minimum	Maximum	Standard
PDGF-BB	27.91	9.70	125.03	3.66	0.04	44.28	4.04	108.66	5.43
FGF basic	62.15	38.14	139.10	2.46	0.04	71.94	12.10	154.97	5.39
G-CSF	81.47	12.32	619.04	21.76	0.02	269.28	0.89	5573.46	227.87
GM-CSF	1.91	0.60	16.95	0.72	0.11	2.19	0.60	17.09	1.17
IFN-γ	65.57	29.72	103.37	2.43	0.38	63.58	1.34	101.78	4.69
IL-2	0.27	0.17	10.61	0.33	0.17	2.07	0.19	9.47	0.57
IL-4	1.74	0.91	6.04	0.15	0.03	2.63	0.31	7.77	0.34
IL-7	1.42	0.57	222.44	5.42	0.27	1.77	0.85	77.33	3.75
IL-9	22.54	9.20	136.74	2.89	0.15	26.82	0.64	97.87	3.51
IL-13	4.30	0.93	17.11	0.57	0.73	4.84	0.93	18.41	0.76
IL-15	77.96	4.50	475.08	16.37	0.04	199.85	4.50	506.56	32.46

## Data Availability

Data are available upon request from the corresponding author.
